# The Detoxification Effects of Melatonin on Aflatoxin-Caused Toxic Effects and Underlying Molecular Mechanisms

**DOI:** 10.3390/antiox13121528

**Published:** 2024-12-13

**Authors:** Chongshan Dai, Daowen Li, Tony Velkov, Jianzhong Shen, Zhihui Hao

**Affiliations:** 1 National Key Laboratory of Veterinary Public Health and Safety, College of Veterinary Medicine, China Agricultural University, Beijing 100193, China; 2 Technology Innovation Center for Food Safety Surveillance and Detection (Hainan), Sanya Institute of China Agricultural University, Sanya 572025, China; 3 College of Animal Science and Veterinary Medicine, Tianjin Agricultural University, Tianjin 300392, China; 4 Department of Pharmacology, Biodiscovery Institute, Monash University, Parkville, VIC 3052, Australia

**Keywords:** AFB1, detoxification effects, melatonin, molecular mechanism, oxidative stress

## Abstract

Aflatoxins (AFTs) are a form of mycotoxins mainly produced by *Aspergillus flavus* and *Aspergillus parasiticus*, which are common contaminants in various agricultural sources such as feed, milk, food, and grain crops. Aflatoxin B1 (AFB1) is the most toxic one among all AFTs. AFB1 undergoes bioactivation into AFB1-8,9-epoxide, then leads to diverse harmful effects such as neurotoxicity, carcinogenicity, hepatotoxicity, reproductive toxicity, nephrotoxicity, and immunotoxicity, with specific molecular mechanisms varying in different pathologies. The detoxification of AFB1 is of great importance for safeguarding the health of animals and humans and has increasingly attracted global attention. Recent research has shown that melatonin supplementation can effectively mitigate AFB1-induced multiple toxic effects. The protection mechanisms of melatonin involve the inhibition of oxidative stress, the upregulation of antioxidant enzyme activity, the reduction of mitochondrial dysfunction, the inactivation of the mitochondrial apoptotic pathway, the blockade of inflammatory responses, and the attenuation of cytochrome P450 enzymes’ expression and activities. In summary, this review sheds new light on the potential role of melatonin as a potential detoxifying agent against AFB1. Further exploration of the precise molecular mechanisms and clinical efficacy of this promising treatment is urgently needed.

## 1. Introduction

Mycotoxins are secondary metabolites produced by certain filamentous fungi such as *Aspergillus parasiticus* and *Aspergillus flavus* and concerningly have been commonly found to contaminate milk, food, Chinese herbal medicine, and grain crops, resulting in multiple detrimental effects on both humans and animals [1]. Currently, there are more than 500 mycotoxins reported worldwide, including ochratoxin A (OTA), aflatoxins (AFTs), zearalenone (ZEN), ochratoxin, deoxynivalenol (DON), T-2 toxin, HT-2 toxin, and fumonisins (FBs) [2,3]. Due to climate change and advancements in detection techniques, the detection rate of mycotoxins is increasing steadily. For example, a recent review indicated that the current detection rate of mycotoxins in global foods or grains is approximately 60–80%, compared to approximately 25% before 1985 [3]. AFTs possess potent toxicities and have been suggested to be linked to multiple chronic diseases, such as neurodegenerative disease, cancer, cardiovascular disease, immunological dysregulation, and chronic liver disease [4,5,6,7,8,9]. A report from the World Health Organization (WHO) showed that about 21,575 foodborne illness cases (including 19,455 deaths) were positively associated with exposure to ATFs worldwide in 2010 [9]. Clearly, mycotoxin contamination of agricultural sources is a critical global public health and safety concern.

**Figure 1:**
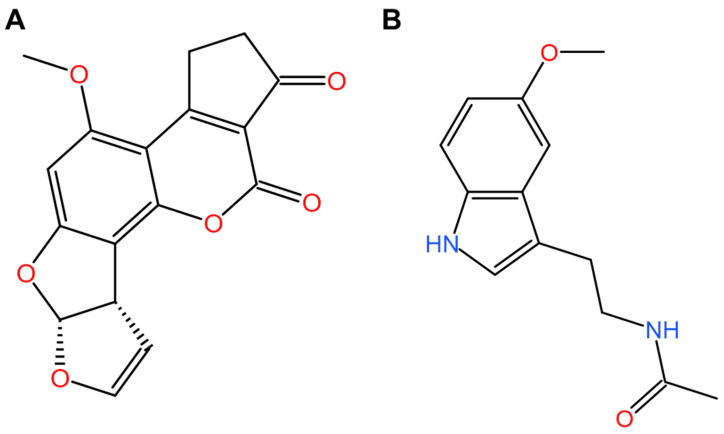
The chemical structure of aflatoxin B1 (AFB1); (**A**) and melatonin (**B**).

According to current reports, aflatoxin B1 (AFB1; Figure 1A) is considered the most toxic one among 21 members of the AFT family [2,3]. It was reported that exposure to exposure could impair child growth [10] and be potently hepatotoxic [11,12,13], cardiotoxic [14], neurotoxic [13,15], nephrotoxic [12,13,16,17,18], immunosuppressant [13,19,20], reproductive toxicity [21], teratogenic [22], and genotoxic [23]. Epidemiological studies have demonstrated that exposure to AFTs is positively associated with liver and gallbladder cancers [24,25]. The International Agency for Research on Cancer has classified AFB1 and its metabolite aflatoxin M1 (AFM1) as Groups I and IIB carcinogens, respectively [26,27].

In mammals, the metabolism and biotransformation of AFB1 mainly take place within liver tissues. Consequently, the liver serves not just as a crucial detoxification organ but also as a toxic target organ of AFB1 [2,28]. This process has been demonstrated to involve various metabolic enzymes such as amino oxidases, epoxide hydrolases, alcohol dehydrogenases, ketone reductases, CYP450s, aldehyde reductases, and monooxygenases [29,30]. Primarily, AFB1 undergoes metabolic processes to form aflatoxin L (AFL) through a reduction reaction, leading to transformation into aflatoxin P1 (AFP1) by O-dealkylation-mediated oxidative reactions or conversion to exo-AFB1-8,9-epoxide (AFBO), aflatoxin M1 (AFM1), AFB2, and AFQ1 through the hydrolytic reaction catalyzed by CYP450s, amino oxidases, epoxide hydrolases, alcohol dehydrogenases, and monooxygenases [29,30]. The molecular mechanisms of AFB1-induced toxicity have been widely studied. AFB1-induced toxic effects are complex and context-dependent [28,31,32,33]. In overview, AFB1 toxicity largely involves cellular redox imbalance, inflammation, DNA damage, autophagy, cell apoptosis, pyroptosis, and necroptosis [2,14,34,35,36,37,38].

Furthermore, a recent study discovered that ferroptosis, a novel form that was defined as iron-dependent programmed cell death, is involved in AFB1-induced cytotoxicity and rat liver injury [39]. Multiple signaling pathways, including solute carrier family 7 member 11 (SLC7A11) [13,40], NLR family pyrin domain-containing 3 (NLRP3) [2,13], p53 [41], nuclear factor erythroid 2-related factor 2 (Nrf2) [2,13,42,43], mitogen-activated protein kinase (MAPK) [44], transforming growth factor-β (TGF-β) [13,24], mammalian target of rapamycin (mTOR) [2,13,45], adenosine 5′-monophosphate (AMP)-activated protein kinase (AMPK) [46], Toll-like receptor 4 (TLR4) [2,13,42,47], nuclear factor-kappa B (NF-κB) [2,13,42,48], phosphatidylinositol 3 kinase (PI3K)/protein kinase B (Akt) [2,49], mitochondrial apoptotic [50], and Wnt/β-catenin [51] pathways, were identified in the process of AFB1-induced cell death [2,13,24,40,42,43,45,47,49]. These signaling pathways are considered crucial targets for toxicity intervention.

Over the past few decades, researchers have made significant progress toward identifying the detoxifying effects of numerous compounds and nutritional supplements such as curcumin, quercetin, baicalin, licochalcone A, and lycopene [18,39,40,52,53]. Notably, recent studies have demonstrated the strong protective effects of melatonin (Figure 1B), a hormone responsible for regulating the sleep–wake cycle, on AFB1-induced harmful effects in mammals [47,54,55,56,57,58,59,60,61,62,63,64,65,66,67,68,69]. It was also reported that melatonin processes diverse biological activities, including anti-inflammatory responses, antioxidant, free radical scavenging, anti-aging, cardiovascular protection, anti-cancer, neuroprotection, immunoregulation, and anti-microbial activities [70,71,72]. Accordingly, melatonin supplementation protects against AFB1 toxicity by suppressing the inflammatory response, eliminating ROS production, upregulating the activities of antioxidant enzymes, and reducing mitochondrial dysfunction and apoptotic cell death [47,55,57,58]. Importantly, a series of clinical trials demonstrated the relative safety of melatonin supplementation in both humans and animals [73,74,75,76]. In China, the United States, and Europe, melatonin supplements have all been approved for commercial non-prescription availability due to their various beneficial effects on neurodegenerative, age-related, and cardiovascular diseases [77,78]. This timely and comprehensive review aims to summarize the detoxifying effects of melatonin against AFB1 toxicity and the contemporary understanding of the underlying molecular mechanisms.

## 2. Biological Activities of Melatonin

**Figure 2:**
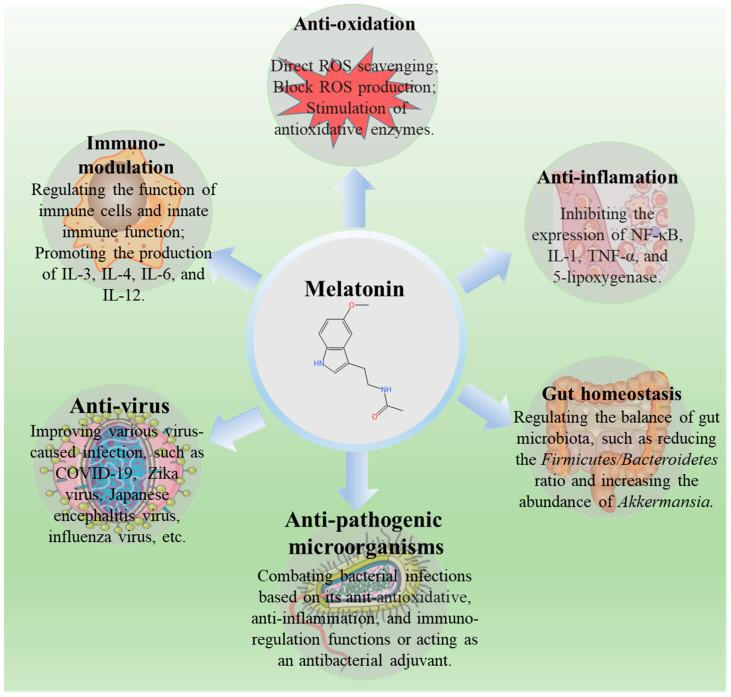
An overview of the application of melatonin in multiple diseases in humans and animals.

Melatonin, chemically known as N-acetyl-5-methoxytryptamine, was first found and identified in 1958 [79]. Belonging to the indoleamine class, melatonin is primarily synthesized from the essential amino acid tryptophan within the pineal gland in the hypothalamus of mammals. Over the past 65 years since its initial discovery, extensive research has enhanced the understanding of melatonin’s biosynthesis, secretion, metabolism, physiological and pathological functions, and regulatory mechanisms [80,81]. Melatonin is also synthesized in a mammal’s heart, bone marrow, skin, gastrointestinal tract, and lymphocyte tissues [82]. In the 1990s, melatonin was proven to be a highly potent hydroxyl radical scavenger, and this finding was considered the starting point of extensive studies on melatonin supplementation as an antioxidant [82,83,84,85]. Currently, melatonin has diverse applications in agriculture, animal production, medicine, and nutritional supplements [86,87,88]. Melatonin has been employed in human and veterinary medicine for treating or preventing neurodegenerative diseases, cardiovascular diseases, infectious diseases, tumors, and chronic metabolic diseases based on its antioxidant, immune regulator, anti-inflammatory, antibacterial, gut microbiota regulation, and antiviral properties (Figure 2) [89,90].

The mechanisms of melatonin-mediated cellular protection are complex and context-dependent. It involves multiple signaling pathways, including PI3K/Akt [73,80,81,88,91,92,93,94], JAK/STAT3 [95], PGC-1α [96,97], peroxisome proliferator-activated receptor (PPAR) [98], SLC7A11 [99], PINK1/Parkin [100], glycolytic [80,88,91,92,101,102,103], NLRP3 [104], mTOR [94], AMPK [100], cAMP/PKA [105], p53 [106], TGF-β [107], p21 [108], Nrf2 [104], NF-Κb [109], MAPK [110], mitochondrial apoptotic [111], GRP78 [101], and PERK [101] pathways, which play critical roles in apoptosis, pyroptosis, ferroptosis, or cuproptosis. Recent studies have highlighted that melatonin supplementation can protect animals or humans from drug-induced toxicity (such as colistin, cisplatin, and gentamicin), mycotoxin poisoning (like T-2 toxin and deoxynivalenol), and exposure to environmental pollutants (such as decabromodiphenyl ether, iron, cadmium, and copper) [102,112,113,114,115,116]. In vitro and animal experiments have further demonstrated that melatonin supplementation can provide effective protection for AFB1-induced reproductive toxicity, liver injury, cardiotoxicity, gastrointestinal toxicity, neurotoxicity, nephrotoxicity, and immune toxicity [55,57,62,63,117]. Notably, recent studies have shown that melatonin exhibits anti-infective effects against bacteria and viruses, as well as perturbing the spread of drug resistance [90,118,119]. A detailed discussion of the specific molecular mechanisms underlying these protective effects will be presented in the subsequent sections.

## 3. Melatonin’s Protective Role Against AFB1 Toxicity and Its Underlying Molecular Mechanisms

Melatonin supplementation offers detoxifying and protection against AFB1 both in vitro and in vivo. The primary molecular mechanisms underlying this cellular protection involve the suppression of ROS production and oxidative stress damage, the reduction of inflammatory responses, the prevention of apoptosis, and the modulation of AFB1 biotransformation. Table 1 provides a summary of melatonin’s protection against AFB1 toxicity, and the detailed discussions are further elaborated in subsequent sections.

### 3.1. Roles of Oxidative Stress

Oxidative stress is a well-recognized mechanism in the toxic pathology of various adverse environmental agents, such as heavy metals, veterinary drugs, organic compounds, and mycotoxins [121]. Reactive nitrogen species (RNS) and reactive oxygen species (ROS), including singlet oxygen (^1^O_2_), superoxide anion (O_2_^−•^), hydroxyl radical (OH•), nitrous acid (HNO_2_), nitric oxide (NO), nitrosyl anion (NO^−^), peroxynitrite (ONOO^−^), nitrotyrosine, and hydrogen peroxide (H_2_O_2_) [122,123], initiate oxidative stress. Under physiological conditions, the intracellular RNS or ROS produced during cell respiration are mostly eliminated by the cell’s self-defense antioxidant system, which comprises cellular antioxidants (such as vitamin C, selenium, and reduced glutathione [GSH]) and antioxidant enzymes (such as glutathione peroxidase [GPX], catalase [CAT], glutathione S-transferase [GST], and superoxide dismutase [SOD]) [2,13]. These antioxidants and antioxidant enzymes maintain the lower levels of ROS or RNS within cells [124]. Disruption of the balance between the antioxidant defense system and the oxidative processes will lead to ineffective elimination of normally generated ROS, resulting in their accumulation within cells and facilitating lipid peroxidation, DNA damage, as well as harm to organelles (e.g., endoplasmic reticulum, mitochondrion, peroxisome, lysosomes, and nucleus), finally culminating in cell death [124].

Numerous studies have indicated that exposure to AFB1 interferes with the antioxidant system, leading to excessive ROS and RNS production in mammalian cells. This process triggers oxidative stress damage and other signaling cascades, such as mitochondrial dysfunction and caspase activation, ultimately resulting in cell death [2,5,13,35,50,125,126,127,128,129,130,131,132,133,134,135]. Melatonin has been reported to possess strong radical-scavenging abilities due to its unique structure, i.e., the N-C=O structure in the C3 amide side chain [136]. It directly scavenges ROS (e.g., H_2_O_2_, ^1^O_2_, OH•, and O_2_^−•^), chelates transition metal ions (e.g., Fe^3+^ and Cu^2+^), and prevents the oxidation of low-density lipoprotein and DNA oxidative damage [137,138,139,140]. In vivo, melatonin’s ability to prevent antioxidant injury is often several times stronger than that of vitamins C and E, which are also effective detoxifying agents for AFB1 [136]. For example, Meki et al. discovered that melatonin supplementation at a dose of 5 mg/kg body weight for 8 weeks effectively reduced the levels of intracellular malondialdehyde (MDA), a marker of lipid peroxidation resulting from AFB1 exposure in rat livers [65,68]. It also significantly increased the levels of GSH, GPX, glutathione reductase (GR), and glutathione-S-transferase (GST) [65,68].

In another study, it was reported that oral administration of melatonin at 5 mg/kg body weight per day for 6 weeks significantly inhibited AFB1-induced ROS production and MDA (a classic biomarker of lipid peroxidation [124]) levels and significantly increased the activities of SOD in rat heart tissues [55]. Similarly, using a porcine oocyte cell culture model, Cheng and colleagues found that the supplementation of melatonin at 0.1, 10, and 100 μM produced a dose-dependent inhibition for AFB1-induced cytotoxicity and apoptosis by inhibiting ROS production and increasing GSH levels [57]. Several studies also found that melatonin supplementation effectively inhibited the production of NO and nitrotyrosine in AFB1-treated liver and stomach tissues of rats [56,62,65,68]. Moreover, this inhibition may be associated with the inhibitory effects of melatonin on the expression of NF-κB/inducible nitric oxide synthase (iNOS) signaling cascade response [141]. These studies confirm that melatonin supplementation can directly inhibit ROS and RNS production and enhance the endogenous antioxidant system, finally improving the cell’s resistance to AFB1.

Nrf2, a housekeeping gene in response to oxidative stress, has been shown to be involved in the process of AFB1-caused cytotoxicity and tissue toxicity [36,142,143]. Nrf2 is a crucial transcription factor that directly or indirectly governs the expression of over two thousand genes, which are involved in various biological processes, including redox-reduction regulation, anti-inflammatory response, antioxidative stress, phase II detoxification enzyme systems, xenobiotic metabolism, and drug detoxification [144,145,146]. Numerous studies have reported that AFB1 can selectively inhibit the transcriptional activation of Nrf2, leading to the reduced expression of its downstream antioxidant and detoxification genes, such as *GST*, *GPX*, *SOD*, *CAT*, and *heme oxygenase-1* (*HO-1*), which ultimately exacerbates cytotoxic effects [125,131,147]. Notably, melatonin has been identified as a potential modulator of Nrf2 [148]. Multiple studies showed that melatonin exerts potent antioxidant, anti-inflammatory, neuroprotective, liver-protective, and cardiovascular protective effects via the activation of the Nrf2 pathway [104,148,149,150]. For instance, Han et al. found that melatonin supplementation effectively reduced chromium-induced lung injury in rats and mouse lung epithelial MLE-12 cells by inducing the Nrf2 pathway by activating the PGC-1α pathway [151]. Kang et al. demonstrated that melatonin treatment at 400 μM of murine macrophages (Raw264.7 cells) and human alveolar epithelial cells (A549 cells) effectively activated the Nrf2/HO-1 pathway, then led to a significant reduction in LPS-induced cell pyroptosis [150]. Consistently, studies have shown that the supplementation of melatonin can markedly upregulate the levels of GST, SOD, CAT, and GPX activities, which are regulated by the transcriptional activity of Nrf2 in response to stress, following to protect cells or tissues against AFB1 exposure-induced oxidative damage [57,68]. These findings suggested that the Nrf2 pathway plays a prominent role in melatonin’s protection effects against AFB1-induced oxidative damage.

**Figure 3:**
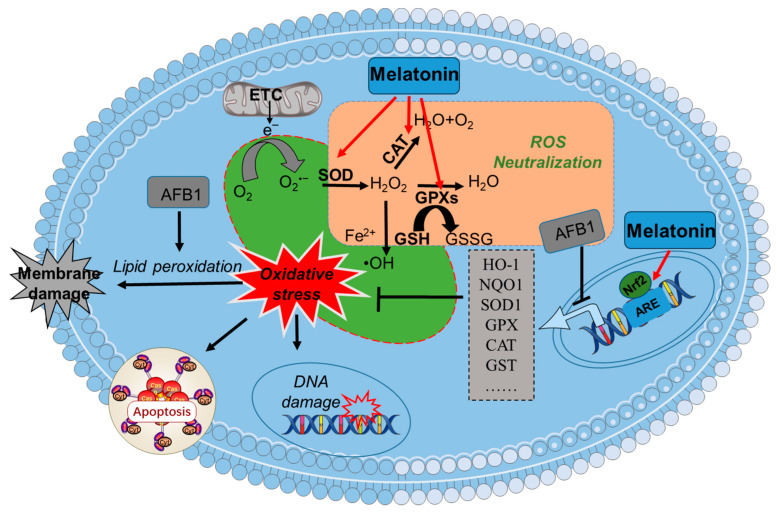
A proposed molecular mechanism of melatonin’s protection against AFB1-caused oxidative damage. ARE—antioxidant response element; CAT—catalase; Nrf2—nuclear factor erythroid 2—Related factor 2; NQO-1—quinone oxidoreductase 1; GST—glutathione S-transferase; ROS—reactive oxygen species; SOD—superoxide dismutase; GPXs—glutathione peroxidases; HO-1—heme oxygenase-1; ETC—electron transport chain.

In conclusion, melatonin supplementation provides a strong protection against AFB1-induced oxidative stress (Figure 3). It may be attributed to its modulation of the Nrf2 pathway.

### 3.2. Inflammatory Response

A link between exposure to AFB1 and the development of significant inflammatory reactions is well established, and prolonged AFB1 exposure can lead to various chronic ailments such as liver cancer, chronic enteritis, and neurodegenerative disorders [152,153,154]. Furthermore, studies have observed that AFB1 treatment can markedly decrease lymphocyte and spleen weights and lower IFN-γ and IL-2 levels in mouse spleen tissues, indicating immunosuppression [19,155]. Importantly, the immunosuppressive effect induced by AFB1 is a significant contributor to the host’s susceptibility to various viruses, such as Epstein–Barr virus, influenza virus, hepatitis B virus, and hepatitis C virus or produces a synergistic damage effect with the virus to host [144,156]. These immunotoxicity and inflammatory responses triggered by AFB1 exposure involve multiple signaling pathways, including the cell cycle arrest, MAPK, NF-κB, Toll-like receptor 4 (TLR4), PKC, Wnt/β-catenin, and NLRP3 pathways [2,153,157,158,159,160,161].

The immune regulatory and anti-inflammatory functions of melatonin in vitro and in vivo have been extensively studied [162,163,164,165]. The mechanism through which melatonin exerts its anti-inflammatory effects is complex. One of the earliest studies suggested that melatonin regulates various transcription factors, such as NF-κB, hypoxia-inducible factor (HIF), and Nrf2, to exert its anti-inflammatory actions [71]. Additionally, melatonin has been found to inhibit lipoxygenase (LOX) and cyclooxygenase (COX), which are two crucial enzymes involved in the inflammatory response [166,167]. Notably, NF-κB is an important transcription factor that controls the expression of multiple genes, such as inducible nitric oxide synthase (iNOS), IL-6, TNF-α, IL-1β, and so on, which are related to innate and adaptive immune function. Melatonin has been shown to inhibit the activation of NF-κB [168]. For example, Yan et al. found that melatonin supplementation via oral administration at 5 mg/kg body weight per for six weeks (i.e., 42 days) could significantly decrease the expression of phosphorylation (p)-NF-κB in the heart tissues of AFB1—treated rats [55]. Akinrinmade et al. showed that melatonin supplementation (intraperitoneal injection, at 10 mg/kg body weight) for seven days significantly reduced AFB1 exposure-induced acute inflammatory response, which was evidenced by the decreases in the reduced hemorrhages and leucocytic and lymphocytic infiltration in the liver and intestines of rats and the decreased level of inflammatory markers such as IL-1β and TNF-α in the serum samples [56,59]. Melatonin has also been shown to eliminate the increases in iNOS and NO levels in tissues exposed to AFB1 [56,62]. These studies indicated that the inhibition of NF-κB plays a critical role in understanding the molecular mechanisms of melatonin’s protection against inflammatory response caused by AFB1 exposure.

The NLRP3 inflammasome plays a vital role in the process of AFB1 exposure-caused inflammatory response, and its activation leads to the secretion of inflammatory markers such as IL-18 and IL-1β [36]. Yan and colleagues found that the supplementation of melatonin can significantly decrease the expression of NLRP3, ASC, caspase-1, and IL-1β proteins and the interaction of NLRP3 and ASC in the heart tissues of AFB1-treated rats, indicating that the inhibition of NLRP3 inflammasome activation contributes to the protective effects of melatonin [55]. Furthermore, the inhibitory effects may partly depend on its antioxidant activity [55]. In addition, it was reported that the targeted inhibition of the TLR4 pathway by melatonin (i.e., significantly reduces the expression of TLR4 and MyD88 mRNAs) could also contribute to its anti-inflammatory effects against AFB1 [47]

It was reported that AFB1 exposure can lead to an imbalance in gut microbiota composition [47,169]. For example, Ye and colleagues showed that AFB1 exposure significantly increased the intestinal Lactobacillus, Bacteroides, and Parabacteroides levels and caused colonic barrier dysfunction and cell pyroptosis in the livers of mice [169]. Liu et al. found that oral melatonin supplementation significantly decreased the abundance of Firmicutes and increased the abundance of Bacteroidetes, then reduced the levels of LPS in the serum and liver samples of mice, finally effectively improving ameliorating inflammatory response caused by AFB1 exposure, although the precise molecular mechanisms remain unclear [47].

**Figure 4:**
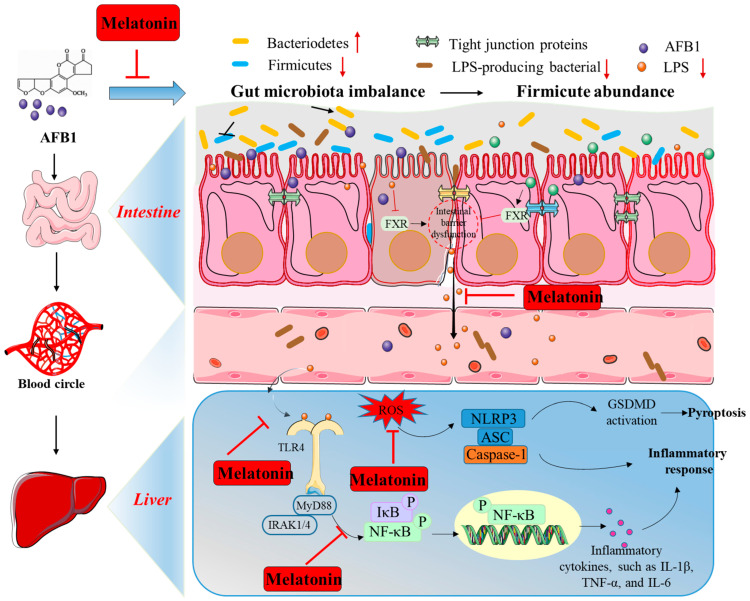
AFB1 triggers gut microbiota imbalance and inflammatory response, as well as the hypothetical modulation by melatonin. AFB1—aflatoxin B1; NF-κB—nuclear factor kappa-B; ROS—reactive oxygen species; NOD-like receptor thermal protein domain associated protein 3; IRAK4—interleukin 1 receptor-associated kinase 4; TLR4—Toll-like receptor 4; ASC—apoptosis-associated speck-like protein containing CARD; IκB—an inhibitor of NF-κB; MyD88—myeloid differentiation factor 88; IL-1β—interleukin-1β; LPS—lipopolysaccharides; TNF-α—tumor necrosis factor-α; IL-6—interleukin-6; NLRP3—NLR family pyrin domain-containing 3; GSDMD—gasdermin D.

In short, as depicted in Figure 4, melatonin supplementation appears to provide protection against the inflammatory response triggered by AFB1 by inhibiting NF-κB, NLRP3, and TLR4 signaling pathways and rebalancing the gut microbiota. However, the precise molecular mechanisms through which melatonin mitigates AFB1-induced inflammation are not fully understood, and further research is warranted in this area.

### 3.3. Mitochondrial Dysfunction and Apoptosis

Exposure to AFB1 can result in the reduction of mitochondrial DNA, the loss of mitochondrial membrane potential, the dysfunction of respiratory function, impaired biosynthesis, and the irregularity of electron transfer [170,171,172,173]. Their toxic effects indicate that mitochondrial dysfunction plays a critical role during AFB1-caused cytotoxicity. AFB1 exposure can also initiate the mitochondrial apoptotic pathway and then induce cell apoptosis [53,170,174,175,176,177]. The Bcl-2 family of proteins, such as Bcl-2, Bax, Bid, and Bak, could primarily regulate the formation of mitochondrial outer membrane permeabilization (MOMP) and then control the initiation of the mitochondrial apoptotic pathway. Subsequently, MOMP could trigger the irreversible release of intermembrane space proteins such as cytochrome C (CytC), then result in the upregulation of caspases’ activities (e.g., caspases-9 and -3) and apoptotic cell death [178,179].

Melatonin possesses the unique ability to be selectively absorbed by mitochondria, which distinguishes it from other antioxidants [180]. Prior findings have highlighted melatonin’s role in preserving mitochondrial homeostasis, with exogenous supplementation proving beneficial in ameliorating mitochondrial dysfunction caused by aging, drug exposure, or toxins [181,182,183,184]. For instance, Xia et al. demonstrated that melatonin supplementation significantly mitigated ochratoxin A (another mycotoxin)-induced oxidative damage and mitochondrial toxicity [185]. Similarly, Cheng et al. reported that melatonin supplementation notably improved AFB1 exposure-induced mitochondrial dysfunction in vitro matured porcine oocytes, consequently inhibiting cell apoptosis [57]. Melatonin supplementation was found to restore mitochondrial mass and number losses from AFB1 exposure by activating mitochondrial biogenesis, shown through the upregulation of mitochondrial fission protein 1 (FIS1), mitofusin 1 (MFN1), and transcription factor B1 (TFB1) mRNAs [57]. This observation reflects an ability to counteract AFB1 exposure-induced mitochondrial dysfunction effectively through mitochondrial biogenesis activation. Furthermore, Meki et al. demonstrated that melatonin administration at 5 mg/kg body weight daily for eight weeks (i.e., 56 days) significantly reduced caspase-3 activities, then effectively attenuated AFB1 exposure-caused cell apoptosis in the liver tissues of rats [65,68]. Excessive ROS generation has been identified as a major factor in inducing apoptosis [186,187]. Yan et al. found that melatonin supplementation via oral administration at 5 mg/kg body weight daily for six weeks (i.e., 42 days) markedly inhibited ROS production, thereby effectively reducing caspase-3 activation and cell apoptosis induced by AFB1 exposure [55,120].

**Figure 5:**
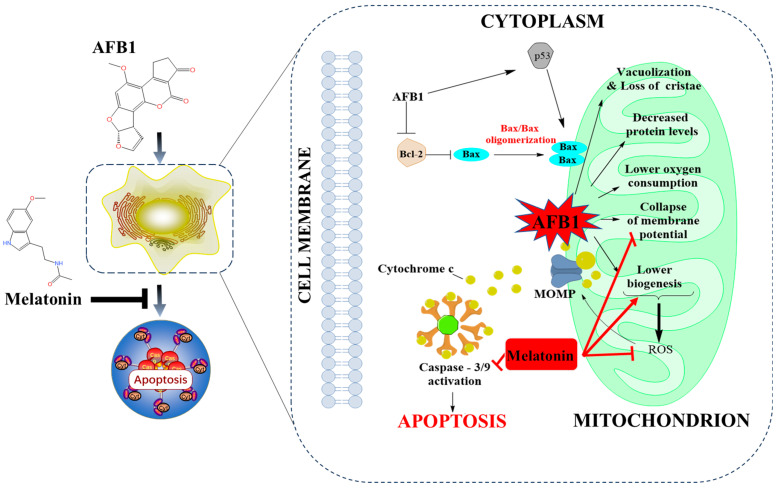
Melatonin’s modulating effects on AFB1-caused mitochondrial dysfunction and cell apoptosis. MOMP—mitochondrial outer membrane permeabilization; ROS—reactive oxygen species; Bax—Bcl2-associated X; Bcl-2—B-cell lymphoma-2; AFB1—aflatoxin B1.

In short, melatonin can inhibit mitochondrial dysfunction and cell apoptosis, following to improve the AFB1-caused harmful effects in mammals. This process may involve the maintenance of mitochondrial membrane potential, promotion of mitochondrial biosynthesis, and inhibition of caspase activity. Figure 5 shows a proposed working model that illustrates the protection mechanisms of melatonin on AFB1 exposure-caused mitochondrial dysfunction and the activation of the mitochondrial apoptotic pathway.

### 3.4. Regulation of Metabolic Enzymes’ Expression and Activity

Several metabolic enzymes, such as CYP3A37, CYP3A4, CYP2A13, CYP2E1, CYP2A6, CYP1A5, CYP1A2, and CYP1A1, have been proven to play key roles in the bioactivation process of AFB1 to AFBO [29,30,188,189,190,191]. AFBO can directly induce cellular toxic effects by directly interacting with DNA, and it was also considered a vital metabolite of AFB1 [192]. Studies have demonstrated that melatonin supplementation exerts strong inhibitory effects on the expression of CYP1A2, CYP1A1, and CYP3A29 enzymes in vitro porcine cumulus cells [57]. Awney et al. found that melatonin supplementation significantly inhibits total CYP450 contents, accompanied by reduced H_2_O_2_ levels, indicating that melatonin’s inhibition of CYP450s depends on its suppression of ROS production [67]. Furthermore, the major pathway for detoxifying AFT metabolites involves AFB1-GSH conjugation [193]. Previous research has shown that melatonin supplementation restores the balance of the GPX/GSH system, consequently maintaining intracellular GSH levels during AFB1 exposure [57]. Moreover, melatonin has been reported to stimulate the expression of GST, an essential detoxifying enzyme that facilitates AFB1-GSH conjugation formation [65,68,194], implying that melatonin’s detoxification effects partially rely on its modulation of the GPX/GSH system.

**Figure 6:**
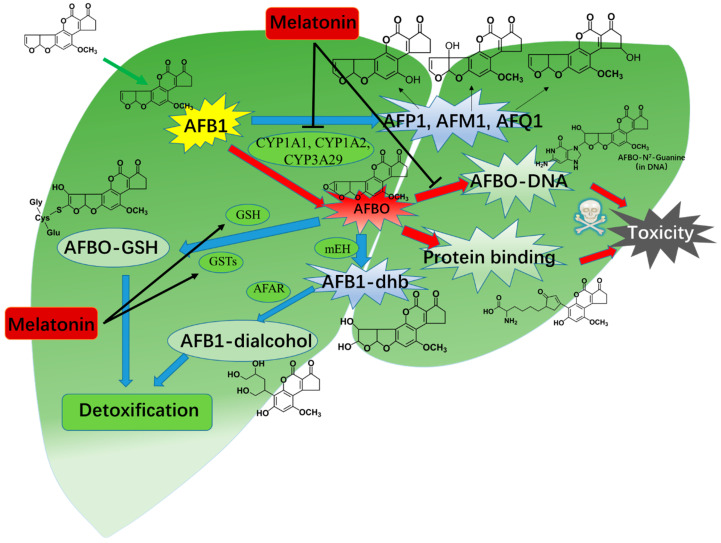
A mechanism pattern diagram of melatonin impact on AFB1′s metabolism pathways. Melatonin could inhibit the activities and expression of CYP450 enzymes, such as CYP1A2, CYP1A1, and CYP3A29. It also promoted the detoxification of AFB1 by maintaining the intracellular GSH levels by regulating the expression of GPXs and GSTs. AFM1—aflatoxicol M1; AFAR—aflatoxin-aldehyde reductase; AFB1-dhb—AFB1-8,9-dihydrodiol; AFBO—exo-AFB1-8,9-epoxide; AFB1—aflatoxin B1; AFP1—aflatoxin P1; CYP450s—cytochrome P450s; AFQ1—aflatoxin Q1; GSTs—glutathione S-transferases; GSH—reduced glutathione; mEH—microsomal epoxide hydrolase.

In short, melatonin supplementation can regulate the AFB1′s bioactivation and detoxification of AFB1 in the liver tissues by regulating metabolic enzymes’ activity and expression. Figure 6 shows a general overview of how melatonin modulates the metabolic pathways that mediate the bioactivation and detoxification of AFB1. However, the exact molecular mechanisms, specifically the way they regulate the expression of metabolic enzymes CYP1As and CYP3A29, are still not clear. It is essential to carry out further verification research in vitro and in vivo.

## 4. Conclusions and Future Directions

AFB1 is an extremely poisonous mycotoxin that is recognized for its cancer-causing effects on both humans and animals. Chronic exposure to AFB1 can result in a range of detrimental effects such as gastrointestinal, neuro-, reproductive, hepato-, immune-, nephro-, and developmental toxicities. In the past few decades, researchers have preliminarily revealed the multiple molecular mechanisms, including the activation of several regular cell deaths (e.g., ferroptosis, autophagy, apoptosis, and necrosis), inflammatory reaction, the induction of oxidative stress and mitochondrial dysfunction during AFB1-induced toxic damage. In detail, these signaling pathways, such as NLRP3, SLC7A11, p53, Nrf2, MAPK, TGF-β, mTOR, AMPK, PI3K/Akt, TLR4, NF-κB, the mitochondrial apoptotic pathway, and Wnt/β-catenin pathways were reported to participate in the regulation of these endpoints caused by AFB1.

Melatonin, which is a hormone in charge of regulating the sleep–wake cycle, has shown its protective functions against the multitude of toxicities caused by AFB1. It involves the inhibition of oxidative stress, the upregulation of antioxidant enzyme activities, the inactivation of mitochondrial apoptotic pathways, and the blockade of inflammatory response. Additionally, melatonin can directly regulate the activities and expressions of CYP450 metabolic enzymes, such as CYP1A2, CYP1A1, and CYP3A29, which reduce the bioactivation of AFB1. Moreover, melatonin can also maintain intracellular GSH levels and enhance the detoxification of AFB1 by modulating the expressions of GPXs and GSTs.

Current data from in vitro and animal experiments indicated that melatonin is a promising detoxification agent for AFB1, although the precise molecular mechanisms remain not completely understood. Therefore, further research is necessary to comprehensively understand the molecular basis of melatonin’s protective effects and evaluate its clinical efficacy. For example, whether melatonin supplementation affects AFB1-induced inflammatory response is associated with its antioxidant capacity and regulatory role in the gut microbiota, and this effect reduces cancer risk caused by AFT exposure. In addition, it is not largely clear whether the key signaling pathway through which melatonin protects against immune suppression and neurotoxicity caused by AFB1 depends on its antioxidant and anti-inflammatory properties or the regulation of gut microbiota. Moreover, current research mainly focuses on animal and cell model studies, while clinical trials are quite scarce on the effectiveness of melatonin against AFB1-related toxic effects, and further verification is needed. AFT-related diseases often occur in conjunction with certain chronic diseases, such as virus infection and diabetes, and it is worth further exploring whether melatonin supplementation can achieve the effect of killing two birds with one stone.

## Figures and Tables

**Table 1:** A summary of melatonin’s protection against AFB1 exposure-induced toxic effects.

Cells/Animals	Treatment	The Regulated Effects	References
Mice	Mice were orally pre-administrated with melatonin at the dose of 20 mg/kg body weight/day. After 2 h, mice were orally administrated with AFB1 at 0.75 mg/kg body weight/day. All mice were treated for 14 days.	Melatonin supplementation markedly attenuated AFB1 exposure-induced imbalance of gut microbiota (i.e., markedly reduced the relative abundance of Firmicutes and the ratio of Firmicutes/Bacteroidetes while increasing the relative abundance of Bacteroidetes), the intestinal barrier impairment, and liver inflammatory response.	[47]
Matured porcine oocytes	Porcine oocytes were pretreated with melatonin at the doses of 0.1, 10 and 1000 μM, then co-treated with AFB1 at the final concentration of 10 μM during oocyte maturation on subsequent preimplantation development.	Melatonin supplementation reduced AFB1 exposure, inhibiting the maturation and development of oocytes via inhibiting oxidative stress and the bioactivation of AFB1 and promoting mitochondrial replication and mitochondrial biogenesis in oocytes.	[57]
Male Sprague-Dawley rats (8-week-old)	Rats were orally treated with melatonin at 5 mg/kg body weight per day with or without AFB1 treatment at 0.3 mg/kg body weight per day for 6 weeks.	Melatonin supplementation significantly attenuated AFB1 treatment-induced myocardial injury in rats by inhibiting oxidative stress, inflammatory response, and apoptosis through the inhibition of ROS production and the inaction of caspase-3, NLRP3, and NF-κB pathways.	[55,120]
One-day-old Ross PM3 breed chicks	Chicks were administrated and fed via a diet containing 150 or 300 ppb AFB1. Meanwhile, chicks were orally administrated with melatonin at 10 mg/kg body weight daily for 21 days.	Melatonin supplementation significantly attenuated AFB1 exposure-induced oxidative stress and apoptosis in the liver and renal tissues by decreasing the levels of MDA and increasing the levels of GSH in the liver and renal tissues.	[62]
Male rats	Male rats were administrated intraperitoneally with melatonin at 20 mg/kg per day with or without AFB1 at 50 μg/kg per day for 3 weeks.	Melatonin supplementation significantly inhibited AFB1 exposure-induced lipid peroxidation (i.e., MDA levels) in the brain, liver, lung, and testis tissues of rats.	[63]
Wistar albino rats	Rats were treated with melatonin at the dose of 10 mg/kg per day via oral administration for 7 days, along with aflatoxin B1 (via the administered administration) on the 5th and 7th days. At the 24th hour after the last administration, rats were anesthetized and euthanized.	Melatonin treatment significantly reduced AFB1 exposure-induced inflammatory response in the liver and small intestine tissues of rats.	[59]
Male rats	Rats were treated (via oral administration) at 5 mg/kg body weight per day with or without AFB1 at 50 μg/kg body weight per day for 8 weeks.	Melatonin administration attenuated AFB1 exposure-induced oxidative damage in the liver tissues by inhibiting the production of MDA and NO and upregulating GPX and GR activities and GSH levels. In addition, melatonin administration attenuated AFB1 exposure-induced apoptosis in the liver tissues.	[65,68]
Chicks	Newly hatched broiler chicks were fed a diet contaminated by AFTs (i.e., 0.5 or 1 mg AFTs per kg diet) or co-fed with Melatonin at 40 mg per kg diet. All chicks were treated for 20 or 40 days.	Melatonin administration significantly improved AFTs exposure-induced lipid peroxidation and oxidative stress damage in the liver and erythrocytes. It also significantly improved AFTs exposure-induced immunosuppression.	[60]
Rats	Rats were orally administrated with melatonin at 10 mg/kg body weight per day for 7 days with or without AFB1 at 2.5 mg/kg body weight on the 5th day. On the 8th day, serum and tissue samples were collected.	Melatonin administration significantly decreased the levels of serum TNF-α and IL-1β levels and NO contents in the stomach tissues.	[56]
Male rats	Rats were treated with AFB1 at 0.2 mg/kg body weight (via the intraperitoneal injection) with or without melatonin at 0.2 mg/kg body weight per day. After 1, 3 and 6 h of injection, the tissues were collected.	Melatonin administration markedly reduced H_2_O_2_ and cytochrome P450 levels in the rat liver microsome.	[67]
